# The Influence of Fatigued Core Muscles on Head Acceleration during Headers in Soccer

**DOI:** 10.3390/sports6020033

**Published:** 2018-04-11

**Authors:** Stephan Becker, Michael Fröhlich, Jens Kelm, Oliver Ludwig

**Affiliations:** 1 Department of Sport Science, Technische Universität Kaiserslautern, 67663 Kaiserslautern, Germany; michael.froehlich@sowi.uni-kl.de (M.F.); oliver.ludwig@sowi-uni-kl.de (O.L.); 2 Chirurgisch-Orthopädisches Zentrum, 66557 Illingen, Germany; jens.kelm@chirurgie-illingen.de

**Keywords:** heading, fatigue, concussion, soccer, acceleration, kinetics, repetitive head impacts, cumulative exposure

## Abstract

The core muscles play a central role in stabilizing the head during headers in soccer. The objective of this study was to examine the influence of a fatigued core musculature on the acceleration of the head during jump headers and run headers. Acceleration of the head was measured in a pre-post-design in 68 soccer players (age: 21.5 ± 3.8 years, height: 180.0 ± 13.9 cm, weight: 76.9 ± 8.1 kg). Data were recorded by means of a telemetric 3D acceleration sensor and with a pendulum header. The treatment encompassed two exercises each for the ventral, lateral, and dorsal muscle chains. The acceleration of the head between pre- and post-test was reduced by 0.3 G (*p* = 0.011) in jump headers and by 0.2 G (*p* = 0.067) in run headers. An additional analysis of all pretests showed an increased acceleration in run headers when compared to stand headers (*p* < 0.001) and jump headers (*p* < 0.001). No differences were found in the sub-group comparisons: semi-professional vs. recreational players, offensive vs. defensive players. Based on the results, we conclude that the acceleration of the head after fatiguing the core muscles does not increase, which stands in contrast to postulated expectations. More tests with accelerated soccer balls are required for a conclusive statement.

## 1. Introduction

Headers, with the head actively being used for influencing a match, are unique in sports and represent an important strategic action in soccer. In current studies, suspicions are expressed that headers may have a negative impact on the brain [1,2]. The potential danger of headers is compared to sports such as boxing, american football, and ice hockey, all of which demonstrably promote the development of chronic traumatic encephalopathy [3,4]. In particular, the risk of recurring traumatic brain injuries (concussions) is high [5,6]. Concussions are said to result from collisions with players or the ground because they strongly accelerate the head [7,8,9]. In addition to these unintentional impacts, another potential danger lies in the high number of headers, i.e., thousands of active headers in the course of a soccer career [10,11,12]. Caccese and colleagues [8] are talking about cumulative exposure in this case, a function based on force and frequency of impacts. They examined the head acceleration of 23 female college soccer players during matches and training. Depending on the type of header or collision, they measured average accelerations of 26.1 G to 51.3 G [10].

From a sports science point of view, many questions on kinetics and kinematics remain to be answered, which would be quite important for understanding and associated preventive approaches [13]. Fatigue during soccer matches has been a topic of enlarged interest, since the overall intensity of play has increased [14,15]. Research concerning the influence of factors such as fatigue has been rather rudimentary to date, especially its effects when heading a ball in soccer [13]. Current studies show, for example, that a muscular imbalance between neck flexors and extensors is associated with an increased acceleration of the head when performing a header [16]. In addition, the strength potential of the neck flexors and extensors seems to be a predictor for the accelerations to be expected [17]. 

During ball contact, the cervical spine and head are in neuromuscular stabilization and stiffened with the trunk, while the acceleration required for a header is generated by the core muscles [18]. Therefore, it seems reasonable to assume that a decoupling of the head–neck–torso alignment takes place due to fatigue or a low training level of the stabilizing muscles, and that in such cases the head is actively moved toward the ball. This in turn results in a reduction of the accelerated mass used for the force impact (head versus head–neck–torso) [19,20]. An amplified nodding motion could then compensate for the fatigued core muscles, which would be associated with an increased activation of the neck musculature [21].

The aim of this study was to examine the influence of a fatigued core-stabilizing musculature on the acceleration of the head during headers from a jumping and running motion. Additionally, the data collected was to determine whether general differences (before fatigue) exist between headers performed from a standing position, a jumping and a running motion. Moreover, we aimed for a sub-group comparison to identify how acceleration differs between defensive and offensive players and between semi-professional and recreational players.

## 2. Materials and Methods

### 2.1. Subjects

A sample of 68 soccer players was the basis for examining the acceleration of the head during headers using a pendulum header before and after fatigue (68 active players: 4th division to 9th division and active recreational players, 21.5 ± 3.8 years, height 180.0 ± 13.9 cm, weight 76.9 ± 8.1 kg). All test persons had long-term match and header experience. Exclusion criteria were acute or chronic cervical spine issues, traumatic brain injuries (concussions) in the eight weeks before the tests as well as acute injuries, acute infections, or illness. Before the beginning of the study, all subjects were informed about the test design, procedure, and potential risks, and gave their written informed consent. Participation was voluntary and did not involve any financial remuneration. The study was designed and conducted based on the current Declaration of Helsinki guidelines and approved by the ethics commission of the Technische Universität Kaiserslautern [22].

The parameters required for categorizing the sub-groups were gathered by means of a separate questionnaire. The categorization limits were normative (see Table 1). Goalkeepers were excluded from the comparison of offensive and defensive players. Players from the 7th, 8th, and 9th division and goalkeepers were excluded from the comparison of semi-professional and recreational players.

### 2.2. Kinetic Analysis

Head acceleration during ball contact was determined by means of a DTS 3D accelerometer (Noraxon USA Inc., Scottsdale, AZ, USA; 22 × 16 × 7 mm 2.8 gm) attached to the occipital area. It was attached using an individually adjustable rubber band (Noraxon USA Inc.; 1000 × 35 mm). The resulting vector was calculated based on the acceleration values of the x-, y- and z-axis. The moment of highest acceleration within six frames (six frames = 0.03 s; from one frame before the first ball contact to five frames after the first ball contact) was determined following Naunheim et al., who stated that the first 0.015 s after the ball contact is crucial [23]. Two tests each were recorded and averaged.

### 2.3. The Header

**Figure 1:**
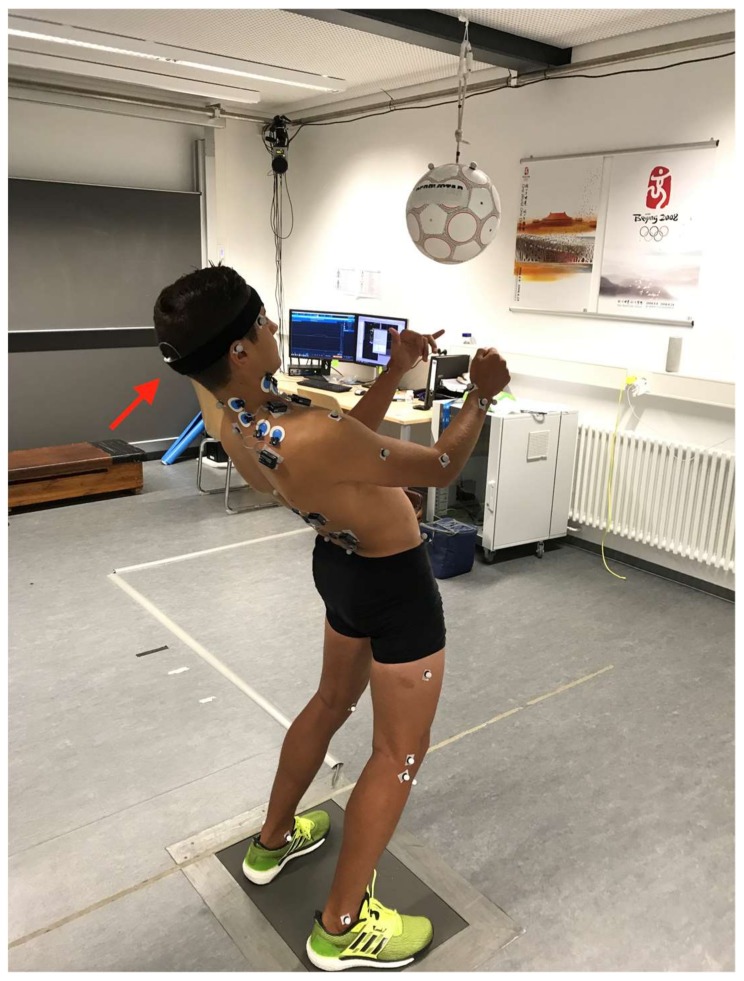
Test design for the variant from a standing position. The red arrow points to the 3D accelerometer in the occipital area.

Three types of headers (from a standing position and from a jumping and running motion) were performed using a pendulum header (Derbystar, Goch, Germany; model: Swing, size: 5, diameter: 22 cm) (see Table 2). In all header variants, the ball was to be headed as powerfully as possible in a horizontal forward direction. The influence of fatigue in the pre-post comparison was determined based on the jump and run header variants. The header from a standing position, which was performed only in the pre-test, was included for an additional comparison between the three header variants and two sub-group comparisons (see Figure 1). In order to familiarize themselves with the pendulum header and to minimize learning effects, the test persons performed three trial headers for each variant before the actual test.

### 2.4. Treatment

**Figure 2:**
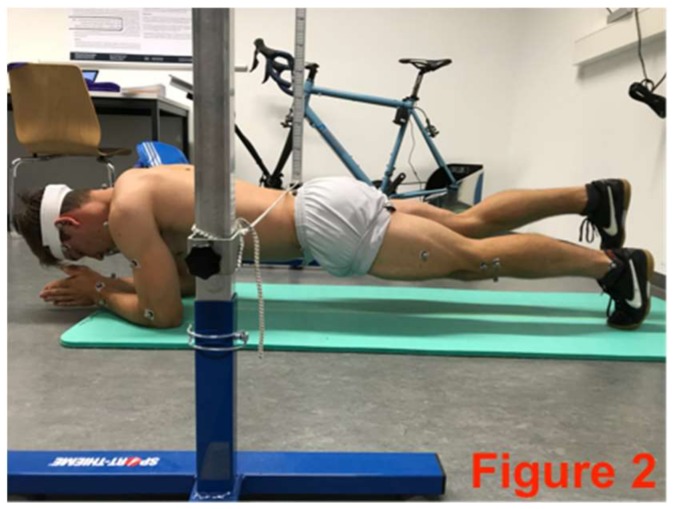
Plank with alternating leg lifts.

**Figure 3:**
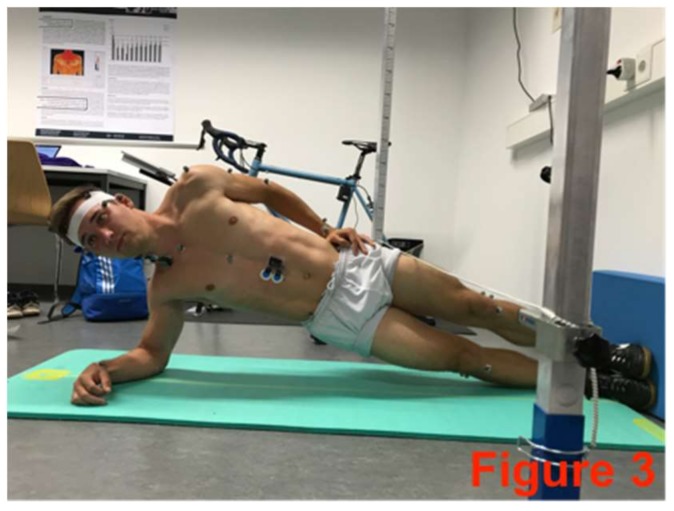
Right-side plank with pelvis drop and lift.

**Figure 4:**
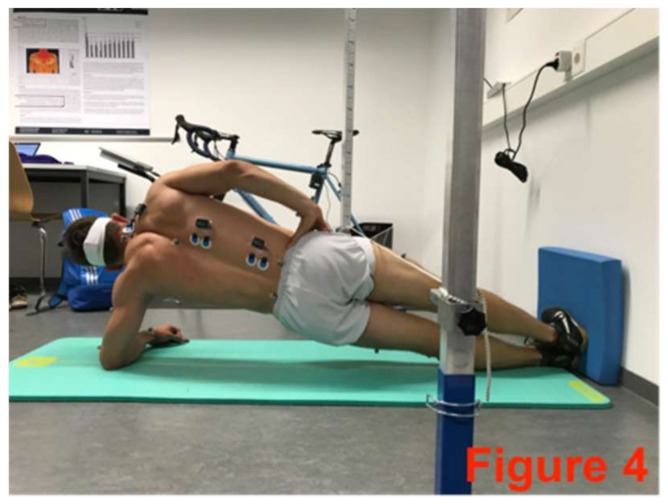
Left-side plank with pelvis drop and lift.

**Figure 5:**
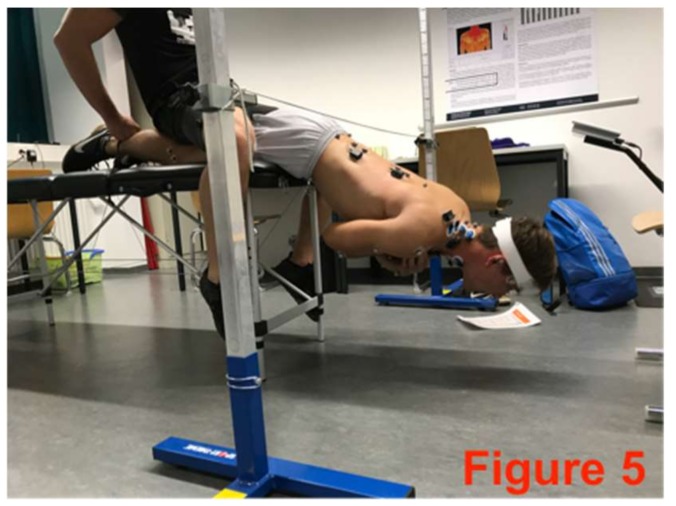
Dynamic hyperextension.

**Figure 6:**
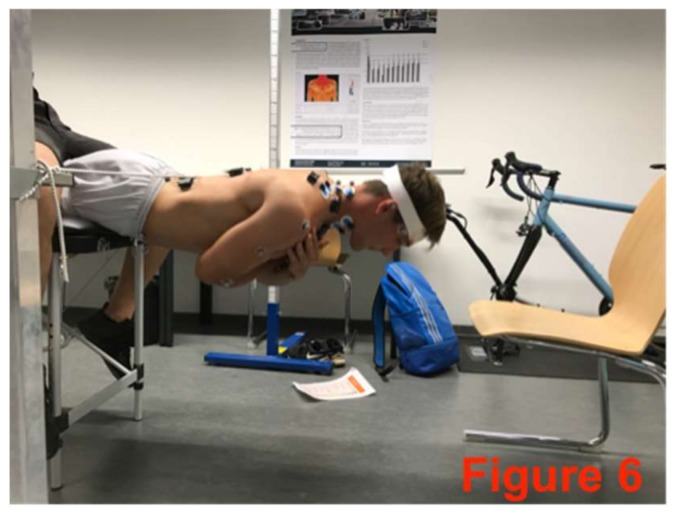
Static hyperextension.

**Figure 7:**
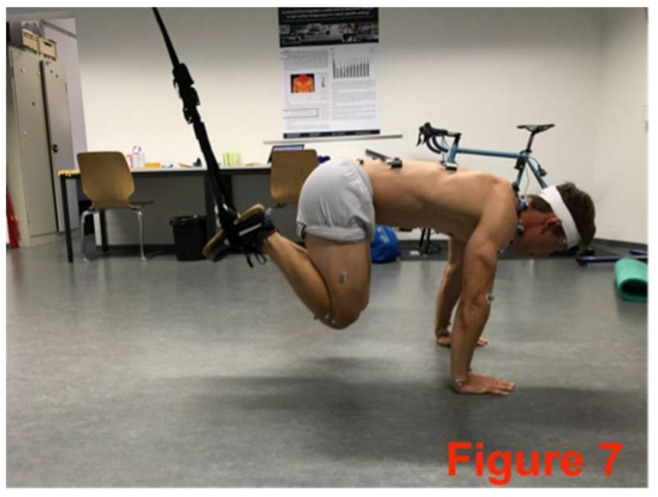
Sling: plank crunch.

The core-stabilizing musculature was fatigued directly after the pre-test based on an extended version of the Bourban test. The first four exercised (Figure 2, Figure 3, Figure 4 and Figure 5) correspond to the Bourban test specifications [24,25]. Subsequently, another back exercise (static hyperextension, Figure 6) and an abdominal exercise (Sling: plank crunch, Figure 7) followed. In all, two exercises were each performed for the ventral, lateral, and dorsal muscle chains:

1. Plank with alternating leg lifts (Figure 2)       (Bourban test)
2. Right-side plank with pelvis drop and lift (Figure 3)  (Bourban test)
3. Left-side plank with pelvis drop and lift (Figure 4)  (Bourban test)
4. Dynamic hyperextension (Figure 5)           (Bourban test)
5. Static hyperextension (Figure 6)            (Bourban test extension)
6. Sling: plank crunch (Figure 7)             (Bourban test extension)

All exercises were performed in one set up to the point of subjective complete exhaustion [26]. Measured by the Borg rating of perceived exertion scale (RPE scale), the perceived exertion was estimated [27]. The effectiveness of the treatment was checked during a separate electromyographical examination with subsequent Fast Fourier Transformation. If the standardized movement amplitude or speed of the test exercise was no longer met, the set was stopped. The movement speed was controlled by means of a metronome and the movement amplitude was controlled using spanned ropes. All exercises were performed in one set with a 1-min break in between them. The post-test was conducted 1 min after the fatigue treatment.

### 2.5. Statistics

The following results are stated as mean values ± standard deviation and 95% confidence intervals. To check pre-post effects (jump and run variants), the *t*-test for dependent samples was applied. The normal distribution was verified by means of the Shapiro–Wilk test. The significance level was set to *p* < 0.05. Effect sizes (Cohen’s *d*) were also calculated and values of 0.20, 0.50, and above 0.80 were considered small, medium, and large, respectively [28]. The statistical evaluation was executed using IBM SPSS (SPSS Version 24.0 for Macintosh, Chicago, IL, USA). Additionally, one-way ANOVA was applied to verify the mean differences of the stand, jump, and run variants as well as two sub-group comparisons. Variance homogeneity was analyzed by means of the Levene test.

## 3. Results

The acceleration of the head between pre- and post-test caused by the fatigue of the core-stabilizing muscles was reduced by 0.3 G in jump headers and by 0.2 G in run headers (see Table 3).

In the post-test, a significant difference (F = 47.67, *p* = 0.000) was identified between the three header variants standing (*N* = 68, 6.0 ± 1.1 G), jumping (*N* = 67, 5.6 ± 1.1 G), and running (*N* = 64, 7.3 ± 0.9 G). The post hoc Scheffé test showed significant differences between the standing and running variants (*p* = 0.000; *d* = 1.29) and between the jumping and running variants (*p* = 0.000, *d* = 1.69).

In the sub-group comparison between offensive and defensive players, no significant difference between the standing, jumping, and running variants could be detected. This also applies to the acceleration values in the sub-group comparison between recreational and semi-professional players (Table 4).

## 4. Discussion

The aim of this study was to examine the influence of a fatigued core-stabilizing musculature on the acceleration of the head during headers from a jumping and running motion. Furthermore, we investigated the potential general differences (before fatigue) between stand, jump and run headers as well as the differences between different players’ positions and skill levels.

The significance of the core muscles for the head–neck–torso alignment for reducing the strain during headers has already been emphasized by several teams [10,29,30]. The stiffer the head–neck–torso alignment, the larger the accelerated mass and the less the resulting acceleration of the head [31]. Hip and trunk extensors provide the arched body tension required, and the trunk flexors are essentially responsible for the acceleration of the head–neck–torso system toward the ball [30,32,33]. Therefore, a compensation mechanism for fatigued core muscles is conceivable. It would counterbalance the reduced activity of the core muscles (particularly the trunk flexors) by an increased activity of the neck muscles, i.e., an increased nodding motion [21]. It needs to be taken into account that this should actually result in dissolution of the head–neck–torso segment stiffness, which, in turn, could lead to increased acceleration of the head. This would increase the potential danger of head injuries [29,34,35]. An increased acceleration of the head should be recorded after fatigue in this case.

However, we were not able to identify an increased acceleration of the head after core muscle fatigue in our test setting. There even was a significant reduction during headers from a jumping motion (*p* = 0.011) and a reduction during headers from a running motion (*p* = 0.067). These results coincide with the results of other tests [13,36]. Two explanations are possible. On the one hand, the reduced acceleration ability or strength development of the trunk seems to be predominant with a fatigued or weak core musculature. On the other hand, the fundamental test setup in the laboratory environment with the motionless pendulum header may be responsible. A field analysis using a ball machine would probably exhibit higher head accelerations and thus higher strain [10,33].

The comparison results of the header variants correspond to the results of earlier tests [18]. In comparison with the standing and jumping header variants, the header from a running motion correlates with a significantly increased acceleration. A general statement on the difference between the standing and jumping variants seems difficult because numerous factors, such as ball flight curve, ball speed, and timing significantly influence the result. In particular, timing and the coordinately more complex movement pattern are considered the reason for the lower degree of acceleration in the header variant from a jumping motion.

No statistically relevant differences were identified in the sub-group comparison between defensive and offensive players. Despite the different requirement profiles of those groups, the motion technique does not seem to differ significantly, which corresponds to the current findings by Caccese and colleagues [10]. Similar results were observed in the sub-group comparison between semi-professional and recreational players. Nevertheless, the averaged data for the recreational players show a reduced acceleration of the head in all three variants compared to the semi-professional players (standing: −0.5 G, jumping: −0.4 G, running: −0.4 G). Presumably, technical superiority effectuates quicker acceleration of trunk and ball. Subsequent analyses should include the strength of the neck flexors for a more detailed comparison. 

This study showed that fatigued core muscles influence the execution of headers in soccer. In contrast to expectations postulated in literature that the acceleration of the head should increase due to a reduced head–neck–torso alignment, the acceleration of the head actually decreases after fatigue [29,34,35]. Use of the pendulum header and the associated elimination of numerous potential disturbance variables (such as ball speed, ball flight curve, initial position, opponents, teammates, etc.) enable a relatively isolated examination of the header. It remains to be clarified whether the acceleration of the head really increases through the reduced head–neck–torso alignment with accelerated balls [37,38]. A preventive approach, for which evidence is yet to be found, would be additional strengthening of the neck flexors and extensors [6,39]. 

The limitations of the present examinations are the use of a motionless pendulum header in a laboratory setting. The high degree of standardization leads to the limited transferability of the results to the accelerations occurring in actual soccer matches. Therefore, the study results’ generalizability is limited. The large degree of heterogeneity in the sample also needs to be taken into account. Additionally, the treatment does not represent soccer-specific fatigue. However, in order to examine the importance of the core muscles and their fatigue while heading the ball, the choice of exercises is limited; soccer-specific fatigue was not the intention of this study.

Furthermore, data evaluation revealed that the acceleration peaks do not necessarily occur at the time of ball contact. Therefore, the time of measurement seems to be important for improved comparability and interpretation. However, this is often neglected or not considered in other studies. Future examinations pertaining to the acceleration of the head should therefore include a more detailed explanation of the time frame that the maximum acceleration is derived from. The state of research in this regard is still deficient. 

Future studies should involve three-dimensional tracking as well as a neuromuscular analysis to get a better understanding on how the core musculature and their fatigue affect the acceleration of the head when heading the ball in soccer. In a different approach, accelerated balls would help to support a general conclusion.

To achieve evidence-based practical applications, further investigations are necessary. So far, the assumption is that an additional strengthening of the neck flexors and extensors, which is not common in soccer, might play an important role in protecting the head from high impacts during headers in soccer. Moreover, the importance of the core musculature for heading, not only for known abilities such as agility, should be underlined [13,40,41].

## 5. Conclusions

Fatigued or weak core muscles are proven to lead to reduced head acceleration at the point of ball contact, measured in an isolated analysis of the header using a pendulum header. This emphasizes the complexity of the motion sequence and shows that it is wise to distance oneself from premature generalizations and warnings [42]. To be able to better judge the influence of cumulative exposure, it is important to first completely understand the movement pattern at the kinetic and kinematic level, including any potential compensation mechanisms in terms of various influencing factors. Headers from a running motion lead to a significantly higher acceleration of the head than the variants from a standing position or jumping motion. In the sub-group comparison, a significant difference was identified neither for the effect of the player’s position in the game, nor for the degree of expertise of a player.

## Figures and Tables

**Table 1:** Sub-group categorization.

Offensive Players	vs.	Defensive Players	Semi-Professional Players	vs.	Recreational Players
Forwards Offensive midfielders (*N* = 25)		Defenders Defensive midfielders (*N* = 39)	6th, 5th, or 4th division (*N* = 39)		≥10th division hobby players ^1^ (*N* = 13)

^1^ At least 2–4 times a month active and no experiences in divisions above the 10th.

**Table 2:** Initial position, take-off, and ball height for the three header variants.

Test Parameter	Standing	Jumping	Running
Initial position	shoulder-wide stand on a force plate (66 × 60 cm)	shoulder-wide stand on a force plate (66 × 60 cm)	Walk position at a mark in 3 m distance
Jump	-	Jumping with both legs	Jumping with one or both legs
Ball height	Height of the forehead	One ball diameter above the head	One ball diameter above the head

**Table 3:** Statistical mean differences in acceleration between pre- and post-test in G.

Parameter	*N*	Pre ± SD	CI	*N*	Post ± SD	CI	T	df	*p*	*d*
Jump headers	67	5.6 ± 1.1	5.39–5.88	67	5.3 ± 1.1	5.05–5.59	2.624	66	0.011	−0.4
Run headers	52	7.4 ± 0.9	7.11–7.58	52	7.2 ± 1.0	6.94–7.48	1.874	51	0.067	−0.2

**Table 4:** Statistical mean differences in acceleration (G) between the sub-groups offensive (O) vs. defensive (D) and semi-professional (SP) vs. recreational (R).

Parameter	Subgroup	*N*	M ± SD	CI	F	df	*p*	*d*
Stand headers	O	25	5.9 ± 1.0	5.53–6.36	1.77	63	0.189	0.4
	D	39	6.3 ± 1.0	5.96–6.62				
Jump headers	O	25	5.6 ± 1.1	5.17–6.11	0.04	62	0.848	0.1
	D	38	5.7 ± 1.1	5.35–6.04				
Run headers ^1^	O	24	7.4 ± 1.0	6.98–7.81	0.00	35.2	0.991	0.0
	D	33	7.4 ± 0.6	7.18–7.61				
Stand headers	SP	39	6.3 ± 1.0	5.99–6.62	2.83	51	0.099	−0.4
	R	13	5.8 ± 1.2	5.06–6.45				
Jump headers	SP	38	5.8 ± 1.1	5.44–6.16	1.62	50	0.209	−0.4
	R	13	5.4 ± 1.1	4.71–5.99				
Run headers	SP	37	7.6 ± 0.9	7.27–7.84	1.21	48	0.277	−0.4
	R	12	7.2 ± 1.1	6.49–7.94				

^1^ The sub-group classification led to a variance inhomogeneity so that the Welch test was conducted.
